# An efficient simulator of 454 data using configurable statistical models

**DOI:** 10.1186/1756-0500-4-449

**Published:** 2011-10-26

**Authors:** Fredrik Lysholm, Björn Andersson, Bengt Persson

**Affiliations:** 1 IFM Bioinformatics and SeRC (Swedish e-Science Research Centre), Linköping University, S-581 83 Linköping, Sweden; 2 Department of Cell and Molecular Biology, Science for Life Laboratory, Karolinska Institutet, S-171 77 Stockholm, Sweden

## Abstract

**Background:**

Roche 454 is one of the major 2^nd ^generation sequencing platforms. The particular characteristics of 454 sequence data pose new challenges for bioinformatic analyses, e.g. assembly and alignment search algorithms. Simulation of these data is therefore useful, in order to further assess how bioinformatic applications and algorithms handle 454 data.

**Findings:**

We developed a new application named 454sim for simulation of 454 data at high speed and accuracy. The program is multi-thread capable and is available as C++ source code or pre-compiled binaries. Sequence reads are simulated by 454sim using a set of statistical models for each chemistry. 454sim simulates recorded peak intensities, peak quality deterioration and it calculates quality values. All three generations of the Roche 454 chemistry ('GS20', 'GS FLX' and 'Titanium') are supported and defined in external text files for easy access and tweaking.

**Conclusions:**

We present a new platform independent application named 454sim. 454sim is generally 200 times faster compared to previous programs and it allows for simple adjustments of the statistical models. These improvements make it possible to carry out more complex and rigorous algorithm evaluations in a reasonable time scale.

## Introduction

The introduction of 2^nd ^generation sequencing techniques has resulted in a myriad of new large DNA sequencing projects, many of which have posed new challenges for bioinformatics. Roche 454 is one of the major 2^nd ^generation sequencing platforms. The 454 instruments use a type of pyrosequencing chemistry, where the complementary strand is elongated through repeated cycles, each using one of the four nucleotides (*flows*). The complementary strand is elongated in the absence of a terminator, and homopolymer lengths are estimated by the light intensity recorded. As a consequence, homopolymer length uncertainties sometimes occur, especially at long homopolymer stretches [1]. This specific aspect of 454 data poses new challenges for downstream bioinformatic applications, such as assembly and alignment search algorithms [2-4]. Simulation of sequence data is an important and extensively used tool for assessing how bioinformatic applications and algorithms handle sequence data. The first software that was developed for simulation of 454 data was MetaSIM [5], which provided simulated data using the statistical distributions suggested for 454 data by Margulies et al. [1]. However, MetaSIM did not produce the raw data that many algorithms utilize and did not model the 'Titanium' chemistry. As a response to these limitations, Flowsim [6] was created. Flowsim produced 454 raw data and allowed for 'Titanium' reads to be produced. Flowsim accepted parameters that modify a particular setting, but all 454 chemistry generations supported by Flowsim were written in source code, which made small modifications complicated. Flowsim and MetaSIM did not produce detailed information of the simulation run, which would for example allow evaluation of correct homopolymer identification in an efficient manner. In order to overcome these limitations, we now introduce 454sim.

## Methods and implementation

454sim generates simulated 454 data from input sequences in FASTA format. The algorithm models *positive flows *(a flow interpreted as one or more bases) with a normal distribution and *negative flows *(a flow interpreted as no base of that type, *i.e*. a noise flow) with a log-normal distribution [1]. This statistical model was used by both MetaSIM [5] and Flowsim [6], although μ and σ for the distributions were chosen differently. In Flowsim, a degeneration model was also introduced, in which the standard deviation was gradually increased along the sequence. Flowsim also provided improved a calculation of quality values based on the statistical model by which flow-peak values were modelled. Both these improvements were also included in 454sim and all parameters can easily be configured through a text format that describes the statistical model used for both flow-peak simulation as well as degradation of the standard deviation along the simulated read. The statistical models for 'GS20' and 'Titanium' that were present in Flowsim have been migrated to 454sim, while 454sim also contains a model for 'GS FLX' reads.

454sim has been implemented using C++ (compatible with GCC and the Intel compiler) to provide a highly efficient and multi-thread capable application. The program uses the Ziggurat algorithm [7] and the "Mersenne Twist Pseudorandom Number Generator Package" [8] to generate random variables with high speed and accuracy. The 454sim project is available as open source under the GNU General Public License. In order to facilitate modifications, it loads the statistical models for each 454 generation from separate text files. This text format defines a series of parameters in a 'key = value' syntax, which is easy to modify using any text editor. For more information regarding the generation parameter files as well as other available parameters, including their usage and examples, see http://www.bioinfo.ifm.liu.se/454tools/454sim or https://sourceforge.net/p/bioinfo-454sim/.

### Evaluation

In order to evaluate the performance of 454sim, we simulated 1,000,000 'Titanium' reads using both 454sim and Flowsim, with default parameter settings, see Table 1. We did not test MetaSIM as it does not produce simulated raw data. For a 454sim run utilizing only a single thread, a 50x increase in speed was achieved. If 454sim was executed using multiple threads, a 200x increase in speed was achieved. This translated to a reduction from approximately 5 1/2 hours down to just over 6 minutes or 1 minute 39 seconds when using multiple threads, see Table 1.

**Table 1:** 454sim performance evaluation

Application	Reads/sec^(*)^	Wall time	CPU time
454sim 8-threads	10,100	1 min 39 sec	11 min 30 sec
454sim 1-thread	2,730	6 min 6 sec	5 min 59 sec
Flowsim 0.2.7	50.3	5 hrs 31 min 40 sec	5 hrs 30 min 20 sec

Evaluation details for the '454sim' and 'flowsim-0.2.7' binary in simulation of 1,000,000 'Titanium' reads. The simulation was performed on a workstation equipped with an "Intel Core i7 920" processor, HT enabled, running Linux 64-bit. * Reads/sec is derived from wall time.

## Discussion

As the amount of sequence data produced keeps increasing, many downstream bioinformatic programs are already adapted to rapidly process large amounts of reads. In order to establish rigorous methods for the evaluation of for these programs, it is important and useful to also be able to simulate reads efficiently. Furthermore, as 454 data quality can vary, for example between metagenomic and genomic sequencing, it is also important to be able to modify the statistical models by which data is simulated. 454sim was constructed to meet these demands. The statistical models describing the Roche 454 platform chemistries are imported from separate text files. These can easily be modified by editing the file using a text editor. Entirely new models can also be added by creating an additional text file. To further facilitate analysis, 454sim also produces optional detailed output where the simulation of each base is described. This enables new types of evaluation such as correct homopolymer indel identification, which is not possible with previous tools. 454sim is at least two orders of magnitude faster than flowsim and reduced the run-time of a 'Titanium' chemistry simulation from 5 1/2hours down to less than 2 minutes, on an Intel Core i7 920. 454sim is platform independent and available as C++ source code [see Additional file 1] or the project homepage.

## Availability and requirements

**Project name: **454sim

**Project home page: **https://sourceforge.net/p/bioinfo-454sim/

**Operating system(s): **Platform independent

**Programming language: **C++

**Other requirements: **--

**License: **GNU General Public License

**Any restrictions to use by non-academics: **--

## Competing interests

The authors declare that they have no competing interests.

## Authors' contributions

FL has implemented the software and written the manuscript. BP and BA have helped design the study and draft the manuscript. All authors read and approved the final manuscript.

## Supplementary Material

Additional file 1 **Binaries and source code**. The file contains Linux 32 and 64-bit binaries, a Windows 32-bit binary as well as source code. See the enclosed README for more information. Click here for file
